# Fabrication of Nickel Nanostructure Arrays Via a Modified Nanosphere Lithography

**DOI:** 10.1007/s11671-010-9770-3

**Published:** 2010-09-02

**Authors:** Xueyong Wei, Xianzhong Chen, Kyle Jiang

**Affiliations:** 1 Bio Medical and Micro Engineering Research Centre, School of Mechanical Engineering, University of Birmingham, Birmingham, B15 2TT, UK

**Keywords:** Electroforming, Level set method, Nanostructure arrays, Nanosphere lithography

## Abstract

In this paper, we present a modified nanosphere lithographic scheme that is based on the self-assembly and electroforming techniques. The scheme was demonstrated to fabricate a nickel template of ordered nanobowl arrays together with a nickel nanostructure array-patterned glass substrate. The hemispherical nanobowls exhibit uniform sizes and smooth interior surfaces, and the shallow nanobowls with a flat bottom on the glass substrate are interconnected as a net structure with uniform thickness. A multiphysics model based on the level set method (LSM) was built up to understand this fabricating process by tracking the interface between the growing nickel and the electrolyte. The fabricated nickel nanobowl template can be used as a mold of long lifetime in soft lithography due to the high strength of nickel. The nanostructure–patterned glass substrate can be used in optical and magnetic devices due to their shape effects. This fabrication scheme can also be extended to a wide range of metals and alloys.

## Introduction

Ordered nanostructure arrays have attracted considerable attention due to their unique properties and potential applications in such as data storage [1], biosensors [2], catalysis [3], and photonic crystals [4,5]. For example, cobalt nanobowl arrays show enhanced coercivity in comparison with spherical particles due to shape effects [6]. However, how to effectively and economically produce such nanostructures in a large area is still a big challenge, even though electron beam lithography, focused ion beam (FIB), and X-ray lithography have been widely used in nanofabrication due to their high resolution. To this end, nanoimprinting lithography (NIL) and nanosphere lithography (NSL), as two low-cost and high-throughput techniques, have been extensively studied in recent time. A high-quality nanoimprint stamp is used in NIL to implement the imprinting, but the stamp fabrication still relies on the aforementioned techniques that are time-consuming and expensive. In the later technique, a monolayer or multilayer of nanospheres is self-assembled and used as the deposition or etch resistive masks. Now, it has been proven to be a promising alternative to the conventional top-down fabrication techniques. For example, through the interstitial spacing between the nanospheres, materials are deposited onto the substrate by using various deposition techniques, such as chemical vapor deposition (CVD) [7], electroless deposition [8], atomic layer deposition (ALD) [9], and even molecular beam epitaxy (MBE) [10], which is used to deposit single crystals and fabricate quantum dot arrays [11,12]. Nanostructures of various shapes such as chains [13], triangles [14], shells [15], rings [16], rods [17], disks [18], and cups [19] have been fabricated on different substrates. Silicon nanopillars were also fabricated using self-assembled nanospheres as a mask in combination with the reactive ion etching (RIE) [20,21] or deep RIE techniques [22]. Clearly, NSL introduces a controllable way to pattern the substrates but the closely packed nanosphere arrays only work as a shadow mask in most cases and high vacuum chambers are required to perform CVD, e-beam evaporation, sputtering, or ALD, which are expensive and slow processes. In this paper, we present a new scheme to fabricate a metallic master template of ordered nanobowl shape together with a nanobowl structures array–patterned glass substrate. Unlike the reported strategies of using self-assembled polystyrene (PS) nanospheres as mask in literature, a monolayer of PS nanospheres was firstly self-assembled on a gold-coated glass substrate, and then a thin gold film was coated on the top of the PS nanospheres. Followed by nickel electroforming, a nanobowl-shaped template was electroformed on the top PS hemispheres, and shallow nickel nanobowl arrays were grown on the gold surface beneath the bottom PS hemispheres.

## Experimental Details

**Figure 1:**
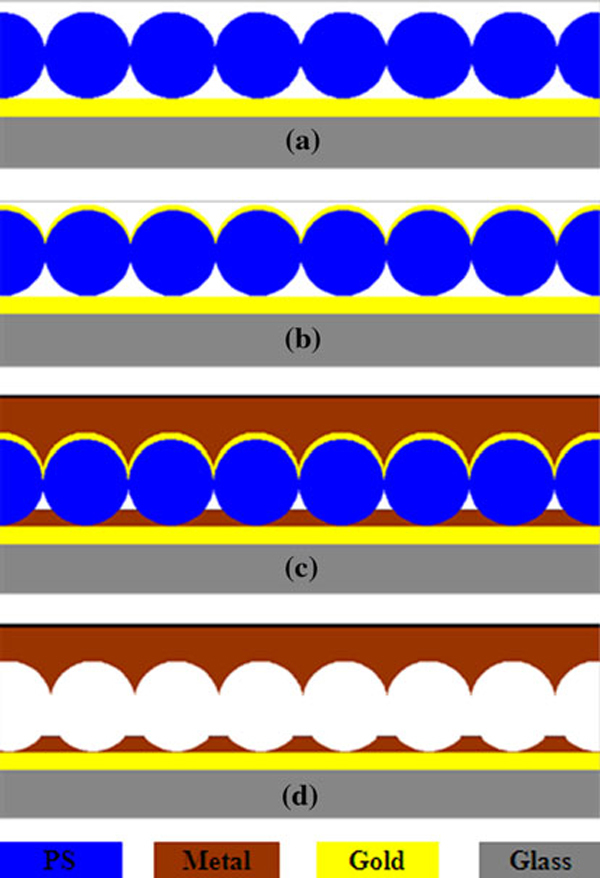
**Schematic process flow of the modified NSL**.

The procedures of our modified NSL is schematically shown in Figure 1. A 2 cm × 2 cm glass slide coated with a thin layer of Au was used as the substrate. The substrate was rinsed in running deionized (DI) water for 1 min, dipped in piranha solution (3:1, H_2_SO_4_:30%H_2_O_2_) for 10 min, and followed by rinsing repeatedly with ultrapure water (18.2 MΩ, Millipore Simplicity). This treatment will create a hydrophilic surface on the gold film to facilitate PS nanosphere assembly. The suspension of PS nanospheres with a mean diameter of 500 nm and a size distribution of 3% (Duke Scientific Corporation) was diluted with ethanol before use. After drying the substrate with nitrogen gas, a 2 μL diluted PS solution was dropped onto the substrate surface using a micropipettor and spread evenly. The single layer of nanospheres on the substrate acted as the template for subsequent processing (Figure 1a). A 50-nm-thick Au film was deposited on the PS surface at a rate of about 16 nm/min in a thermal evaporation system at a pressure of 2 × 10^-5^mbar (Figure 1b). A nickel sulfamate electrolyte was employed to perform electroforming experiments due to its high deposition rate and low stress in the deposits (Figure 1c). A 5 cm × 8 cm pure nickel plate was used as the auxiliary electrode. The pH value and temperature of the electrolyte were maintained at 4.2 and 50°C, respectively. Finally, the nickel sheet was separated from the substrate and etched in tetrahydrofuran (THF) for 10 min to remove the PS nanosphere (Figure 1d).

The microscopic examination of the produced samples was conducted in our focused ion beam (FIB, StrataTM DB 235, FEI) system. In order to understand the pattern formation around the PS nanospheres, a multiphysics modeling of the process was carried out by using a finite element analysis package (Comsol Multiphysics 3.5a). In this model, the mass transport of chemical species was simulated on the base of the Nernst-Planck application mode, and the moving interface between the electrolyte and metal was analyzed by using the level set application mode.

## Results and Discussion

**Figure 2:**
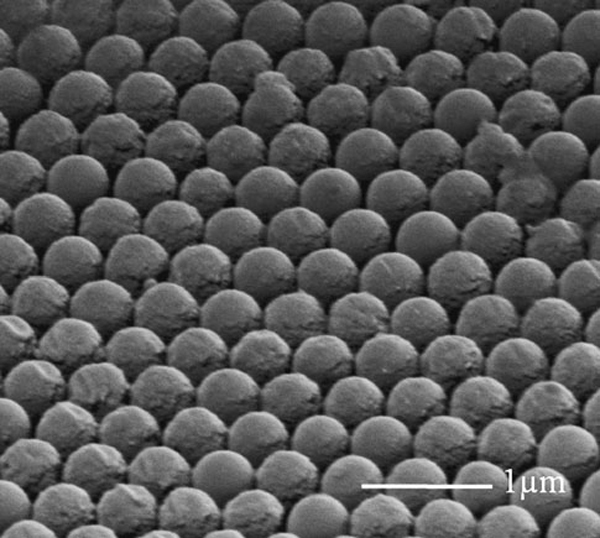
**SEM image of a monolayer PS nanospheres after gold coating**.

Figure 2 shows a scanning electron microscopy (SEM) image of a monolayer of close-packed PS nanospheres. The thickness of Au layer on the PS nanospheres was controlled within 50 nm in order to avoid blocking the openings between PS nanospheres. Experimental results and numerical modeling of gold film thickness on the top nanosphere surfaces indicate that it increases from the contacted points to the vertex of nanospheres as schematically shown in Figure 1b. Hence, the interstitial spacing between PS nanospheres is still left open to allow the electrolyte to reach the substrate, which makes it possible for electroforming to take place in the narrow gaps.

**Figure 3:**
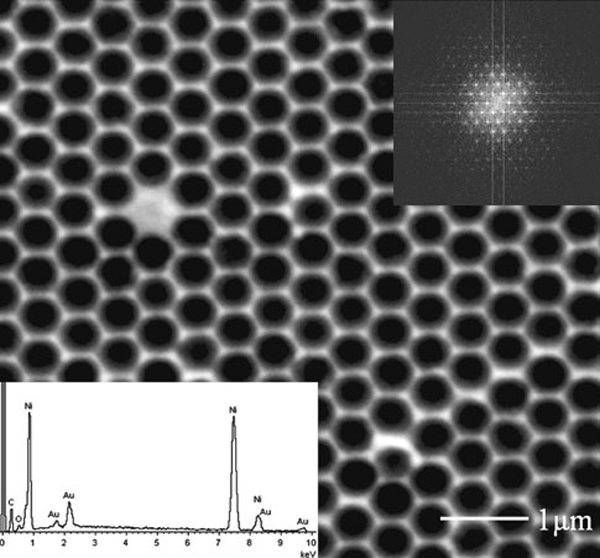
**SEM images of the resultant nickel nanobowls array *top inset*: FFT pattern of the image; and *bottom inset*: EDX spectrum of the nanobowls**.

**Figure 4:**
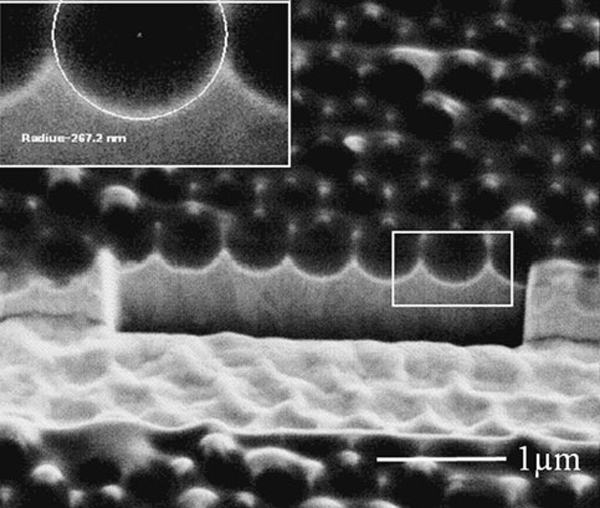
**FIB cross-sectioned nickel nanobowls array (*inset*, the fitted diameter of nickel nanobowl using image processing technique)**.

The resultant nickel master template of a bowl-shaped nanostructure is shown in Figure 3. The PS nanospheres were washed away during the removal process, and EDX spectrum (bottom inset) indicates that the bright regions in the image are gold residuals. The pitch and size of the nanobowl can be adjusted independently by selecting PS nanospheres with different sizes or by decreasing the PS nanosphere size with oxygen plasma etching after the self-assembly process. Fast Fourier Transformation (FFT) technique is an excellent method to study the periodicity of micro- and nanostructures configuration [23]. In this study, the regularity of nickel nanobowls array was analyzed by applying FFT to the SEM images. The top inset in Figure 3 shows that the hexagonal arrangement of nickel nanobowls is quite uniform, and the nickel nanobowls array is of a six-fold symmetry. Figure 4 shows a SEM image of the nickel nanobowl cross-section milled using FIB with an ion beam current of about 100 pA. The nanobowl size was calculated from the SEM images of nanobowl cross-section using an image processing technique. It was found that the curvature radius of the nanobowl is about 267 nm less than the estimated 300 nm, which is due to the misalignment between the cutting line and the center line of the nanobowl and also the image contrast induced edge detection error because each pixel of our SEM images represents approximately 5 nm in length. The profile of the FIB cut nanobowls can be more accurately reconstructed and errors can be minimized by positioning the cutting line through the center of nanobowl and by improving the contrast of SEM images.

**Figure 5:**
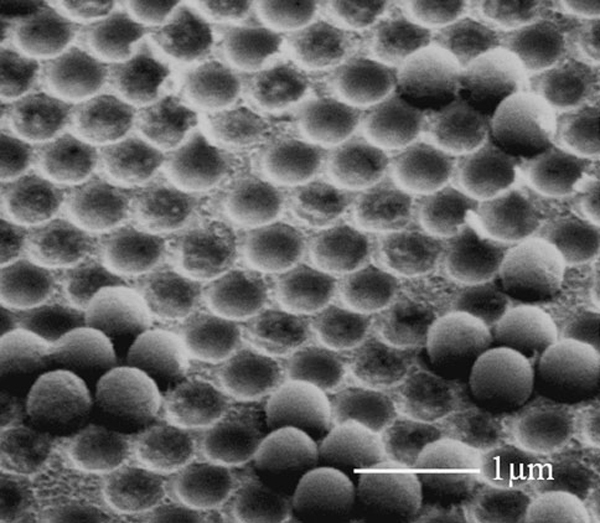
**SEM image of the patterned glass substrate with arrays of shallow nickel nanobowls, where some are still occupied by the PS nanospheres after THF etching**.

The shallow nanobowl structure–patterned gold surface as shown in Figure 5 was also obtained after separating the top layer of master template of nanobowls shape. The flat bottom is due to the contact between PS nanospheres and the substrate. There are still some nanobowls occupied by the PS nanospheres left after THF etching process, which implies that the nanostructures can be used to select nanospheres of similar or smaller sizes for subsequent processing [24]. The SEM characterization shows that the nickel nanobowl structures with the flat bottom are about 100 nm thick. In fact, the thickness of nickel nanostructures growing around the bottom PS nanospheres is determined by the faradic current or the reductive ion flux passing through the interstitials between the nanospheres. When the electroforming process starts, nickel ions are reduced simultaneously on both the top surface of PS nanospheres and the gold-coated glass substrate. However, nickel ions transported to the interstitial spaces are gradually decreased due to the diminishing of opening between the PS nanospheres.

**Figure 6:**
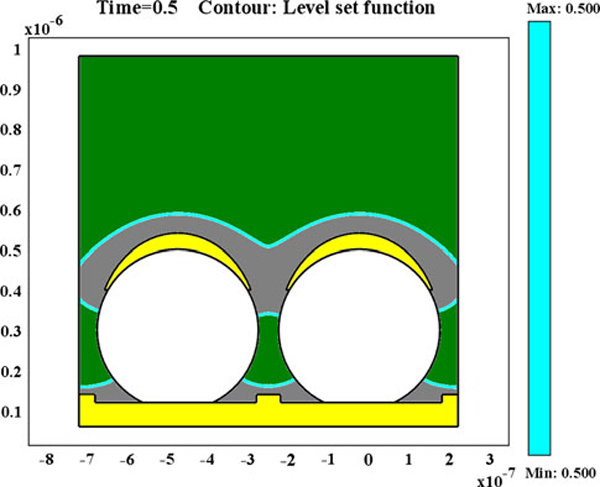
**Multiphysics simulation of the growing nickel surface around the PS nanospheres, where the lines of *light blue* color represent the interface between electrolyte (the *green area*) and electro-deposited nickel (the *gray area*)**.

**Figure 7:**
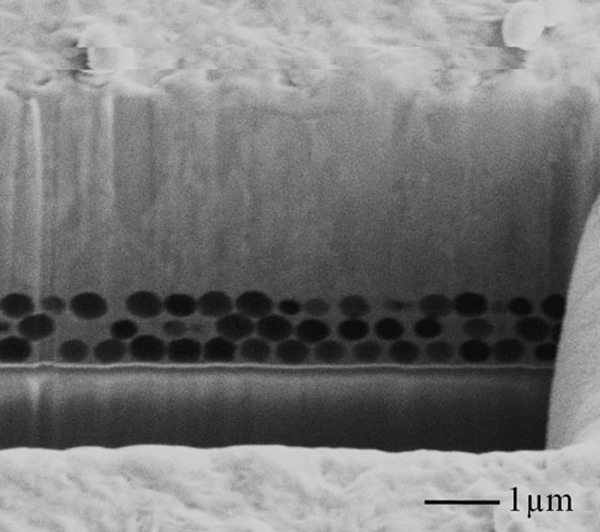
**SEM image of a FIB cross-section on the edge of PS template after nickel electroforming**.

In this study, the multiphysics model based on level set method (LSM) was built up to investigate this metal forming process through tracking the position of the metal and electrolyte interface. The LSM has already been shown to be a powerful method for studying deposition processes and tracking the evolution of the moving interface between deposits and electrolyte [25]. In the LSM, a scalar variable, φ, is defined over the entire computation region, and the set of locations φ = 0.5 in our model (usually, the zero level set φ = 0 is used) defines the position of the interface. Figure 6 shows that the nickel deposits on the top of the nanospheres have merged, and the interstitial between the nanospheres are blocked at *T* = 0.5 s. The fast electroforming rate is due to that a high current density (500 A/m^2^) was used in the simulation to decrease the computation time. However, a low current density (15 A/m^2^) was used in the experiment to avoid the hydrogen bubbles and black deposits. The simulation also reveals that nickel deposits can fill the whole interstitials among the nanospheres once the top openings are not blocked by growing metal; in other words, PS nanopsheres are not closely packed together (simulation results are not shown here). The FIB milled cross-section (Figure 7) shows the complete filling of nickel deposits around the nanospheres on the edge of the PS nanotemplate where multiple layers of PS nanopsheres are self-assembled. Undoubtedly, a porous nickel nanostructural material can be made in this manner by using the close-packed three dimensional nanospheres as template for electroforming.

## Conclusions

In summary, we proposed and demonstrated a modified NSL method to fabricate metallic nanostructures in a simple and inexpensive way. A nickel template of ordered nanobowls array together with nanostructured modified glass substrate with a gold-coated surface was fabricated by combining self-assembly method with electroforming. The nickel nanobowls exhibit a uniform size and smooth interior surface. No shrinkage of the material occurs in this process when the PS template is removed. The electroforming process can be easily controlled by adjusting operating parameters to produce high-density and fine grain–structured metal deposits. Compared with other metal deposition methods as reported in the literature, electroforming is a facile method to fabricate metallic nanostructures at a low-cost and high-throughput [16,18].The choice of deposited materials can be also extended to other metals (e.g. Au, Ag) and alloys (e.g. Fe-Pt, Co-Pt).
